# Editorial: Phytohormones as crucial players in organ abscission

**DOI:** 10.3389/fpls.2025.1716731

**Published:** 2025-10-14

**Authors:** Agata Kućko, Emilia Wilmowicz, Timothy John Tranbarger

**Affiliations:** ^1^ Department of Plant Physiology, Institute of Biology, Warsaw University of Life Sciences, Warsaw, Poland; ^2^ Department of Plant Physiology and Biotechnology, Faculty of Biological and Veterinary Sciences, Nicolaus Copernicus University, Toruń, Poland; ^3^ UMR DIADE, IRD Centre de Montpellier, Institut de Recherche pour le Développement, Université de Montpellier, Montpellier, France

**Keywords:** organ abscission, abscission zone, hormones, signaling, fruit crops

Organ abscission, governed by specialized cell layers called the abscission zone (AZ), is a critical developmental process in plants, modulated by environmental factors and essential for harvest efficiency, crop quality, and agricultural management. Delayed harvesting due to ripening asynchrony exacerbates preharvest drop and uneven yield, placing additional strain on growers. Despite the agricultural importance of understanding abscission, many core mechanisms of molecular and hormonal coordination remain elusive, especially the intricate phytohormone crosstalk that times organ detachment.

This Research Topic brings together five innovative experimental studies alongside two comprehensive reviews that illuminate the crucial role of phytohormones as abscission master regulators, offering new insights into the physiology, signaling networks, and practical control of this process in crop species such as apple, mulberry, pear, oil tea, and cotton. The collective emphasis is made predominantly on integrating multiscale molecular dynamics with practical agricultural perspectives and management techniques. Through the utilization of state-of-the-art approaches – ranging from rigorous field experiments and advanced multi-omics analyses to high-resolution imaging techniques – the assembled studies provide fresh mechanistic insights into organ abscission while offering actionable knowledge applicable to crop yield optimization and quality enhancement.

In the study by Johnson and Farcuch, comprehensive field experiments demonstrate that ethylene (ET)-inhibiting agents aminoethoxyvinylglycine (AVG) and 1-methylcyclopropene (1-MCP) markedly reduce fruit cracking in ‘Ambrosia’ apples, mitigate preharvest drop, and modulate fruit color and ripening dynamics. However, efficacy varies significantly by cultivar and season, as ‘Fuji’ apples exhibit a contrasting response under mid-Atlantic growing conditions. Notably, AVG treatments provide the most potent suppression of ET biosynthesis/signaling gene expression, but also delay red skin coloration by diminishing activity in the anthocyanin biosynthesis pathway. These findings underscore the nuanced balance among ET’s roles in fruit quality, pigmentation, and gene regulation, offering an essential framework for fine-tuning ET management strategies in apple production systems.

Yang et al. further advance the molecular understanding of fruit abscission through an innovative multi-omics exploration of *Morus laevigata* (long-fruited mulberry). By integrating transcriptomic, metabolomic, and proteomic data, these researchers reconstructed a comprehensive gene-metabolite interaction network that orchestrates fruit detachment. A constellation of key transporter proteins and cell wall-modifying enzymes emerged as critical hubs mediating abscission, highlighting the intricate crosstalk between metabolic pathways and transcriptional regulators in governing this vital developmental process. In particular, the authors provided evidence that an increased cell wall-degrading enzyme activity during mulberry fruitlet abscission may result from the combined effects of abscisic acid (ABA) accumulation and auxin signaling activation.

Building on the spatiotemporal dimension of hormonal regulation, Zheng et al. harnessed mass spectrometry imaging coupled with ultrastructural analyses in *Pyrus sinkiangensis Yü* (Korla fragrant pear), revealing the dynamic distribution and opposing actions of phytohormones within the AZ. Their work illuminates how ABA and ET synergistically promote calyx abscission, whereas auxin, gibberellins (GAs), and zeatin exert inhibitory effects. These spatial maps of hormone localization and molecular activity deepen our theoretical understanding and offer practical guidance for precision management of calyx retention – critical for enhancing fruit quality in pear cultivation.

Shu et al. investigated the combined effects of the plant growth regulators thidiazuron (TDZ) and cyclanilide (CYC) on cotton leaf abscission under low-temperature conditions, where defoliation efficiency is challenged. Through transcriptome profiling, these authors demonstrated that the combined treatment enhances leaf abscission by suppressing auxin signaling genes and activating ET and jasmonate (JA) response pathways, accompanied by increased reactive oxygen species (ROS) activity within the AZ. This hormonal and oxidative rebalancing orchestrated by TDZ and CYC provides a robust mechanistic foundation for improving cotton harvest management where low temperatures otherwise impair defoliant efficacy.

Ma et al. delve into immature fruit abscission of *Camellia oleifera*, a key limiting factor for yield in this economically valuable oil-producing tree. Their field data identify August as the peak period for fruit drop, marked by a sharp decline in auxin and JA levels alongside a concurrent rise in trans-zeatin within the AZ. Comprehensive transcriptomic analyses revealed upregulation of ET biosynthesis and signaling genes, as well as factors linked to cell wall degradation and oxidative metabolism. Among the key regulators, the transcription factors NAC DOMAIN CONTAINING PROTEIN 100 (NAC100) and ETHYLENE RESPONSE FACTOR114 (ERF114) emerged as central orchestrators of the hormonal and transcriptional cascades triggering abscission, presenting promising molecular targets for biotechnological or agronomic strategies to reduce premature fruit loss.

Complementing these original experimental studies, two reviews offer expansive conceptual frameworks that bridge core molecular insights with practical applications. Tipu and Sherif emphasize ET’s role as a central hormonal maestro in climacteric fruit ripening, coordinating physiological and biochemical ripening hallmarks - such as color development, softening, sugar metabolism, and aroma production - and interconnecting with auxin, ABA, GA, JA, brassinosteroid (BR), and salicylic acid (SA) pathways to shape complex ripening phenotypes and abscission dynamics. In the review, the authors translate these ET-centered insights into commercial strategies, highlighting the optimal timing and application of growth regulators to enhance fruit quality and marketability in diverse horticultural systems.

Tranbarger and Tadeo examine the metabolic activities within AZs that profoundly influence preharvest and postharvest fruit quality. The authors detail how interplay among ET, auxin, ABA, and JA modulates fruit detachment, affecting critical quality traits – including size, firmness, peel and flesh coloration, and shelf-life extension. The review further explores multilayered hormonal crosstalk within the AZ and the fruit, spotlighting MADS-box transcription factors that form regulatory complexes linking AZ development with ET biosynthesis and ripening pathways. These insights underscore the AZ as a nexus integrating developmental, hormonal, and environmental signals to finely tune fruit detachment and quality.

Collectively, these contributions demonstrate that organ abscission is regulated not by a single linear hormonal pathway but by complex, context-dependent networks involving dynamic phytohormone gradients, intricate gene expression modulation, and environmental cues. The integration of multi-omics technologies, precise imaging methods, and rigorous field trials advances mechanistic understanding while delivering actionable insights for breeding, chemical interventions, precision agronomy, and crop improvement. By placing phytohormones at the center of abscission control, this body of work elevates the field toward a multidisciplinary paradigm in which deep mechanistic knowledge drives the development of resilient, high-yielding, and quality-focused crop systems capable of meeting global food production challenges.

